# Hemostatic net in cervical and facial surgeries: evidence from a systematic review and meta-analysis

**DOI:** 10.1590/acb412326

**Published:** 2026-05-18

**Authors:** Felipe Giraldo Gonçalves, Leonardo Dexheimer da Silva, Emily Caroline da Fonseca Sampaio, Artur de Souza Almeida, Bruno Damico Terada, Guilherme Azeredo Malacarne, Ilan David Friedmann, Miguel Rodrigues Moreira, Rolf Gemperli, Cristina Pires Camargo

**Affiliations:** 1 Universidade de São Paulo – Faculdade de Medicina – Departament of Surgery – Division of Plastic Surgery – São Paulo (SP) – Brazil.; 2 Universidade de São Paulo – Faculdade de Medicina – Laboratory of Plastic Surgery and Regenerative Medicine – São Paulo (SP) – Brazil.

**Keywords:** Rhytidectomy, Hemostasis, Systematic Reviews as Topic, Meta-Analysis as Topic

## Abstract

**Purpose:**

Hematoma remains the most frequent complication of cervicofacial surgeries. The hemostatic net (HN), a transcutaneous suture designed to minimize dead space and bleeding, has shown promise, but pooled evidence of its safety and efficacy is limited. Thus, we conducted this study to clarify the safety profile and complication rates associated with the use of HN in cervical and facial surgeries.

**Methods:**

A systematic search of MEDLINE, Embase, Scopus, Cochrane, and Web of Science was performed through August 2025. Eligible studies involved adults undergoing cervical and/or facial surgery with HN, with no restrictions on suture caliber or spacing between stitches. The primary outcome was hematoma incidence; secondary outcomes included skin necrosis, seroma, infection, persistent skin marks, patient-reported satisfaction, and other adverse events.

**Results:**

Eleven studies including 3,393 patients (2,748 HN; 645 controls) were analyzed. Single-arm meta-analysis of HN cases showed low pooled rates for hematoma (0.6%; 95% confidence interval 0.4–1.0; low certainty), skin necrosis (0.4%; 95% confidence interval 0.1–1.0; low certainty), seroma (1.5%; 95% confidence interval 0.6–4.0; low certainty), infection (0.1%; 95% confidence interval 0.0–25.5), and persistent skin marks (0.6%; 95% confidence interval 0.2–2.1). Comparative analyses showed no significant difference for hematoma (risk ratio = 0.15; 95% confidence interval 0.01–3.20; very low certainty) or seroma (risk ratio = 1.06; 95% confidence interval 0.41–2.75; low certainty). Patient-reported satisfaction data were scarce and nonspecific.

**Conclusion:**

The HN is a safe technique with very low complication rates, though evidence of superiority over standard surgery remains inconclusive due to limited certainty of meta-analyzed data, heterogeneity of surgical procedures, and the paucity of patient-reported outcomes.

## Introduction

Facial and cervical plastic surgeries ranked in 2024 as the most frequently performed group of aesthetic procedures worldwide, surpassing five million cases, mainly through eyelid surgery and rhytidectomy^1^, which restore a youthful appearance by refining contours and correcting facial laxity^2-6^. This surgery alone accounted for over 1.7 million procedures worldwide, with 115,124 procedures performed in the United States of America^1,7^. Cervical and facial surgeries often involve extensive skin detachment. Therefore, postoperative hematoma represents a major clinical concern and remains the most frequent complication, potentially leading to venous congestion, permanent scarring, and flap necrosis if not promptly treated^8-12^.

Despite advancements in perioperative care, including systolic blood pressure control^8^, surgical drains^8,13-16^, compression dressings^15,17-19^, and meticulous intraoperative hemostasis^20-22^, hematoma continues to occur, with incidence reported in 0 to 15% of cases^8,23^. This variability underscores the absence of a consistently effective preventive strategy.

In this context, the hemostatic net (HN), a surgical technique developed by Auersvald et al.^24,25^, has emerged as a novel approach to stabilize tissue planes, minimize dead space, and enhance hemostasis, thereby reducing the risk of postoperative bleeding^8,26-29^. Although early reports suggest favorable outcomes, the evidence lacks pooled analyses to confirm its benefits over established methods and estimate the real-world incidence of complications following this technique^8,27-31^. To address this gap, the present systematic review and meta-analysis evaluated the efficacy of the HN in reducing postoperative complications following facial and cervical plastic surgeries.

## Definition of hemostatic net

The HN, as originally described by Auersvald and Auersvald^24,25^, consists of a continuous transcutaneous suture that anchors the skin flap to the underlying fibroconnective tissue of the superficial musculoaponeurotic system and platysma^24,28,31^. The suture is passed percutaneously in a crisscross fashion, producing an external grid with the characteristic appearance of a fishing net^24,28,31^. This external pattern corresponds to the internal fixation points, which stabilize the flap, obliterate dead space, and provide homogeneous compression across the undermined area^8,24,26-29,32,33^. By limiting shear forces and securing the flap against deeper tissues, the technique enhances hemostasis, prevents fluid accumulation, and thereby reduces the incidence of postoperative hematoma and seroma^8,24,26-29,32,33^.

## Methods

This systematic review and meta-analysis was conducted in accordance with the *Cochrane Handbook for Systematic Reviews of Interventions* ^34^ and the PRISMA Statement guidelines^35^. The protocol was prospectively registered in PROSPERO under the number CRD420251143364.

### Search strategy

A systematic literature search was performed for studies published up to August 2025 in MEDLINE, Embase, Scopus, Cochrane, and Web of Science databases. Search terms combined keywords related to HN in facial or cervical surgeries, including their synonyms and Medical Subject Headings (MeSH) terms.

### Eligibility criteria

#### Inclusion criteria

The authors included studies that met the following criteria:

- Involved adult patients (≥ 18 years old) of both genders, undergoing any cervical or facial surgery;
- Reported the use of an external HN technique, following our previous definition (external transcutaneous running quilting sutures, regardless of suture caliber, timing of removal, spacing between stitches, or specific anatomical application);
- Any follow-up duration was accepted;
- Eligible study designs included randomized trials, observational studies, and case series.

#### Exclusion criteria

The authors excluded the following studies and patient groups:

- Cadaveric or animal studies;
- Studies using quilting sutures other than the external HN;
- Individual case reports, reviews, editorials, and letters without original data;
- Studies with overlapping populations. In cases in which multiple articles reported data from the same patient cohort, we included only the study with the largest sample size. An exception was made when one of the overlapping studies was a controlled trial and another was an uncontrolled study. In these situations, both were included in the systematic review, but only one was included in each quantitative analysis—the larger cohort was used for the single-arm meta-analysis, and the controlled study was used for the comparative meta-analysis, ensuring no overlapping at all.

### Screening and study selection

The initial screening was conducted in Rayyan (Rayyan Systems Inc., Qatar), after deduplication in EndNote Online 20 (Clarivate, PA, United States of America). Two reviewers independently assessed titles and abstracts, with discrepancies resolved by a third reviewer. Full-text articles were also independently reviewed by the same two authors, and disagreements were resolved by the third one. No automation or artificial intelligence tools were applied at any step of the process.

### Endpoints

The primary endpoint was the incidence of postoperative hematoma, while secondary endpoints included skin necrosis, seroma, infection, persistent skin markings, patient-reported satisfaction, and any other adverse events reported by included studies (such as congestion, transient neuromuscular deficits, and other ones). Complication outcomes were collected as defined in the original studies.

### Data extraction

To extract the data from the articles, two independent authors used a predefined protocol. The variables included demographics, surgery details, HN techniques, follow-up, and clinical outcomes.

### Articles quality assessment

Risk of bias was assessed according to study design: randomized controlled trials (RCTs) with the Cochrane RoB 2 tool^36^; non-randomized controlled studies with ROBINS-I^37^; and case series with the Joanna Briggs Institute (JBI) Checklist for Case Series^38^. Two investigators performed all assessments independently, resolving disagreements by discussion and consensus.

### Statistical analysis

For pooled proportion analysis, a single-arm model was conducted using the generalized linear mixed model. Pooled estimates were exposed with their respective 95% confidence intervals (95%CI). The comparative meta-analyses were performed using pooled risk ratios (RR) with 95%CI under random-effects models to account for clinical and methodological variability. Statistical significance was defined as a two-sided *p* < 0.05. Heterogeneity was assessed using the I^2^ statistic, and values ≥ 30% were used as a prespecified threshold to trigger sensitivity analyses (leave-one-out [LOO] and meta-regressions) to explore potential sources of variability. All analyses were conducted in R (version 4.5.0; R Foundation for Statistical Computing, Vienna, Austria) using RStudio (Posit, PBC, Boston, MA, United States of America).

Certainty of evidence was assessed using the Grading of Recommendations Assessment, Development and Evaluation (GRADE)^39^ approach.

## Results

### Study selection and characteristics

**Figure 1:**
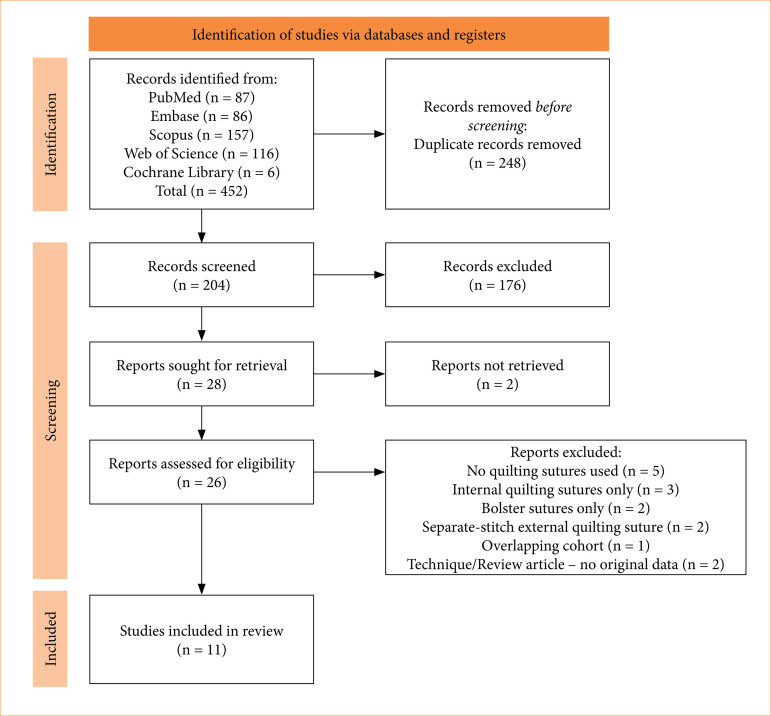
PRISMA 2020 flow diagram for new systematic reviews which included searches of databases and registers only.

Source: adapted from Page et al.^35^.

After conducting the systematic search, 452 articles were initially identified. Following deduplication, 204 studies remained. After screening titles, abstracts, and full texts, 11 studies^6,8,28,29,40-46^ met the eligibility criteria and were included in this review (Fig. 1). Collectively, these studies encompassed 3,393 patients, of whom 2,748 were allocated to the HN group and 645 to the control group. A detailed summary of the baseline characteristics of the included studies is presented in Table 1, and the clinical endpoints reported are outlined in Table 2.

**Table 1:** Baseline characteristics of patients in the included studies.

Study ID	Patients enrolled (N)	Mean age (years old)	Female (N [%])	Smoking history (N [%])	Hypertension (N [%])	Study design	Intervention	Comparator	Surgeries	Drains (Y/N)	Dressings	HN technique	Days to remove hemostatic net	Follow-up
Ismail and Ghoraba^8^	Total: 160; HN: 80; D: 80	45.5	HN: 80 (100); D: 80 (100)	13 (8.1)	NR	RCT	HN	D	Facelift	Yes, D group	NR	3/0 prolene, entering the skin perpendicularly, transfixing at a 45-degree angle into the platysma, and emerging at the same angle approximately 1 cm	2	At least two weeks
Auersvald et al.^28^	Total: 525; HN: 405; D: 120	HN: 55.4 (34–81); D: 55.4 (33–77)	HN: 369 (91); D: 109 (90.8)	HN: 46 (11.3); D: 24 (20)	HN: 28 (6.9); D: 11 (9.2٪	Cohort	HN	D	Facelift	Yes, D group	Occlusive dressing	5-0 mononylon; continuous sutures plunging into the SMAS-Platysma	2	3 months
Janssen et al.^29^	Total: 663; HN: 304; D: 359	62.4 (38–86)	HN: 284 (93.4); D: 327 (91)	NR	NR	Cohort	HN	D	Facelift	Yes, D group	NR	5-0 polypropylene, 90° 1×1 cm	2	At least two days
Auersvald et al.^31^	HN: 1,019	50.6 (16–81)	925 (90.8)	NR	NR	Case series	HN	Not applicable	Facelift and necklift	No	NR	5-0 mononylon; continuous sutures plunging into the SMAS-platysma and emerging 0.8–1 cm from the entry	2–3	NR
Kachare et al.^41^	HN: 8	64 ± 19.6	1 (12.5)	6 (75)	5 (62.5)	Case series	HN	Not applicable	Cervicofacial flap reconstruction	No	NR	5-0 nylon, 1×1 cm	NR	9.3 (1–19) months
O'Daniel et al.^42^	Total: 108; HN: 66; D: 42	NR	NR	NR	NR	Cohort	HN	D	Facelift	Yes, D group	NR	5 0 mononylon, platysma at 45 degrees, 1 × 1 cm	2–3	At least two days
Orra et al.^43^	HN: 63	62.8 ± 7	59 (93.4)	6 (9.6)	13 (20.6)	Case series	HN	Not applicable	Gliding brow lift	NR	NR	4-0 prolene, 1×1 cm	2–3	8.3 (3–54) months
Şibar et al.^44^	Total: 86; HN: 42; control: 44	HN: 50.8 ± 8.3; control: 51.2 ± 8.2	HN: 40 (95.2); control: 42 (95)	NR	NR	Cohort	GBL with HN	Endoscopic mesh lift without HN (control)	Brow lift	NR	NR	4-0 Polypropylene, 0.5 × 0.5 cm	2	HN: 17.3 (13–31) months; Control: 24.7 (13–40) months
Sozer et al.^45^	HN: 337	61 (24–88)	304 (90.2)	NR	NR	Case series	HN	Not applicable	Neck lift	No	None	2-0 PDO, midline application followed by a lateral plication	3	12 months
Viterbo et al.^46^	HN: 124	55.6 ± 7.9	114 (92)	NR	NR	Case series	HN	Not applicable	Gliding brow lift	No	None	4-0 nylon; 0.5–1.0 cm × 0.5–1 (height)	2	17 (3–35) months
Wongkietkachorn et al.^58^	HN: 300	54.2 ± 10.0	275 (91.7)	15 (5)	33 (11)	Case series	HN	Not applicable	Gliding brow lift, facelift, or necklift	No	Gauze and bandages	5-0 prolene	3	At least one week

HN: hemostatic net; D: drains; SMAS: superficial musculoaponeurotic system; NR: not reported; RCT: randomized controlled trial; GBL: gliding brow lift. Source: Elaborated by the authors.

**Table 2:** Clinical endpoints reported in the included studies.

Authors	Hematoma (N)	Skin necrosis (N)	Seroma (N)	Infection (N)	Persistent skin marks (N)	Patient’s satisfaction	Other adverse events
Ismail and Ghoraba^8^	HN: 1; D: 0	HN: 0; D: 0	HN: 7; D:7	HN: 0; D: 2	NR	NR	Congestion: HN: 2; D: 2
Auersvald et al.^28^	HN: 0; D: 17	HN: 3; D: 6	NR	NR	HN: 3; D: 0	NR	Ischemia: HN: 26; D: 11
Janssen et al.^29^	HN: 2; D: 14	NR	NR	NR	NR	NR	NR
Auersvald et al.^31^	7	NR	11	NR	NR	NR	Weakness of the lower lip depressor: 79; salivary fistulae of SMGs: 4; sialoma of SMGs: 2
Kachare et al.^41^	0	0	1	0	0	NR	Swelling: 2
O'Daniel et al.^42^	HN: 0; D: 0	HN: 0; D: 0	HN: 1; D: 0	NR	HN: 0; D: 0	One patient had concerns about the hyperemic appearance of the suture marks at two weeks post-operatively, which was successfully and quickly resolved with a singlepulsed-dye laser treatment	Hyperemia: HN: 1; D: 0
Orra et al.^43^	1	NR	NR	3	3	NR	Temporal fullness: 3
Şibar et al.^44^	HN: 0; Control: 0	HN: 0; Control: 0	HN: 0; Control: 0	HN: 0; Control: 0	1	Global aesthetic improvement scale assessment: HN: 1.79 ± 0.71; Control: 1.32 ± 0.58	Frontal nerve palsy (HN): 1; dog ear on the scalp (HN): 1; mesh irritation (control): 2; unilateral dysesthesia on the scalp (control): 1
Sozer et al.^45^	1	1	4	NR	1	NR	Neck contour irregularity: 10; Residual platysmal or skin laxity: 18
Viterbo et al.^46^	0	0	0	0	NR	Very satisfied: 20٪; satisfied: 49٪; non-satisfied and non-dissatisfied: 24٪; dissatisfied: 7٪; very dissatisfied: 0٪	Recurrence requiring revisional procedures: 6
Wongkietkachorn et al.^58^	2	NR	NR	NR	0	Patients have expressed concerns about the duration required for the HN markings to disappear	Skin irregularities: 2

HN: hemostatic net; D: drains; NR: not reported; SMG: submandibular gland. Source: Elaborated by the authors.

### Primary endpoint

**Figure 2:**
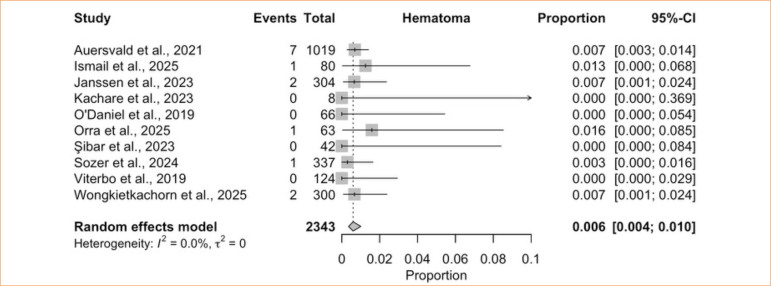
Proportional meta-analysis of hematoma: hemostatic net only.

Source: Elaborated by the authors.

The single-arm meta-analysis of postoperative hematoma included 10 studies^6,8,29,40-46^, totaling 2,343 patients treated with a HN after cervical and/or facial surgery. The pooled incidence was 0.6% (95%CI, 0.4–1.0%; I^2^ = 0%; low certainty) (Fig. 2), with no heterogeneity.

**Figure 3:**
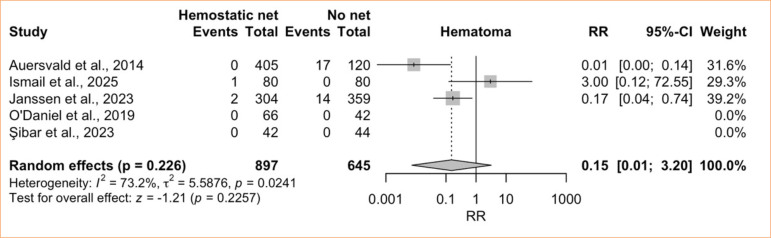
Controlled meta-analysis of hematoma: hemostatic net versus control.

Source: Elaborated by the authors.

**Figure 4:**
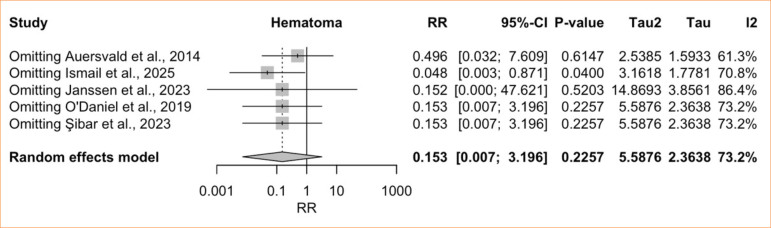
Leave-one-out hematoma: hemostatic net versus control.

Source: Elaborated by the authors.

The comparative meta-analysis comprised five studies^8,28,29,42,44^ with 1,542 patients (897 with HN and 645 without). The initial pooled estimate revealed high heterogeneity and no significant difference between groups (RR = 0.15; 95%CI 0.01–3.20; *p* = 0.23; I^2^ = 73.2%; very low certainty) (Fig. 3). A LOO sensitivity analysis showed that Ismail and Ghoraba’s8 omission yielded a significant effect favoring the HN (RR = 0.048; 95%CI 0.003–0.871; *p* = 0.04), while heterogeneity remained high (I2 = 70.8%) (Fig. 4).

**Figure 5:**
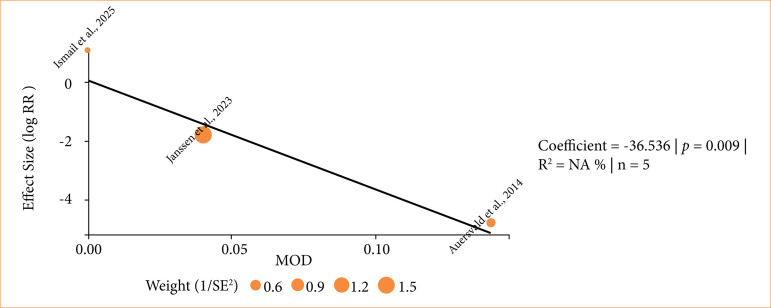
Meta-regression of hematoma: hemostatic net versus control—control-group hematoma incidence as the covariate.

Source: Elaborated by the authors.

Given the absence of heterogeneity in the single-arm analysis, we hypothesized that variability in the comparative analysis stemmed mainly from control arms, in which hematoma rates varied widely. This variability likely reflects differences across studies in perioperative management protocols, surgical technique, and hematoma definitions within the control groups, rather than inconsistency in the performance of the hemostatic net itself. A meta-regression using control-group hematoma incidence (Fig. 5) confirmed a significant association with the outcome (β = -36.5; standard error = 14.1; *p* = 0.009) and reduced the residual variance (τ^2^) to 0, indicating that this factor fully explained the heterogeneity. R^2^ was not computed because the base model’s between-study variance was near 0.

### Secondary endpoints

**Figure 6:**
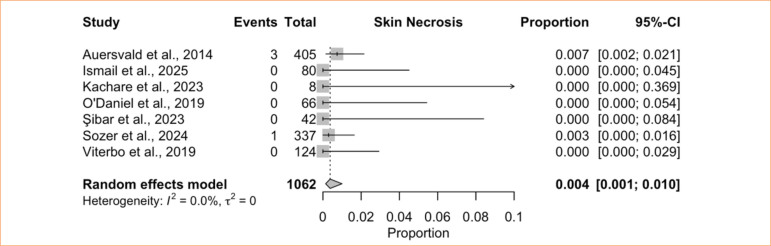
Proportional meta-analysis of skin necrosis: hemostatic net only.

Source: Elaborated by the authors.

**Figure 7:**
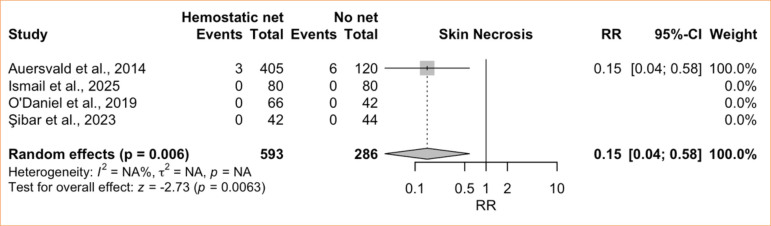
Controlled meta-analysis of skin necrosis: hemostatic net versus control.

Source: Elaborated by the authors.

The single-arm meta-analysis of skin necrosis included seven studies^8,28,41,42,44-46^, comprising 1,062 patients treated with HN. The pooled incidence was 0.4% (95%CI 0.1–1.0%; I^2^ = 0%; low certainty) (Fig. 6), indicating no heterogeneity. The comparative meta-analysis for this endpoint encompassed four studies^8,28,42,44^ with 879 patients (593 with HN and 286 without). Among these, three studies reported zero events in both groups, so only a single study contributed meaningfully to the pooled effect estimate. Consequently, the pooled analysis favored the HN group (RR = 0.15; 95%CI 0.04–0.58; *p* = 0.006; low certainty) (Fig. 7), but heterogeneity statistics (I^2^ and τ^2^) could not be calculated.

**Figure 8:**
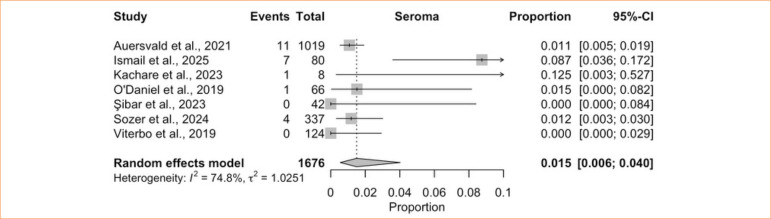
Proportional meta-analysis of seroma: hemostatic net only.

Source: Elaborated by the authors.

**Figure 9:**
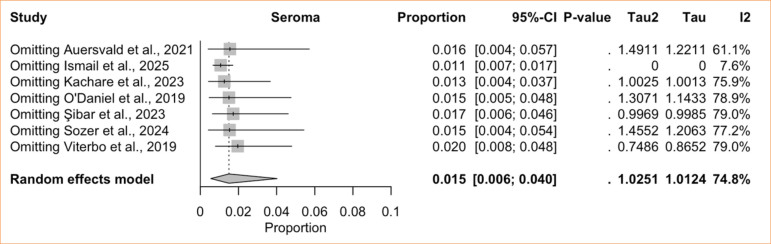
Leave-one-out seroma: hemostatic net only.

Source: Elaborated by the authors.

**Figure 10:**
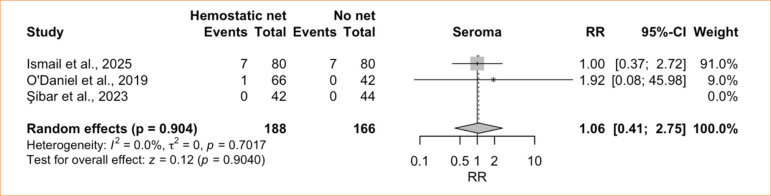
Controlled meta-analysis of seroma: hemostatic net *versus* control.

Source: Elaborated by the authors.

The single-arm meta-analysis of seroma included seven studies^8,40-42,44-46^, comprising 1,676 patients treated with HN. The pooled incidence was 1.5% (95%CI 0.6–4.0%; I^2^ = 74.8%; low certainty) (Fig. 8), with high heterogeneity. Sensitivity analysis using the LOO method revealed that omitting Ismail and Ghoraba^8^ markedly reduced heterogeneity, yielding a pooled incidence of 1.1% (95%CI 0.7–1.7%; I^2^ = 7.6%) (Fig. 9). The comparative meta-analysis encompassed three studies^8,42,44^ including 354 patients (188 with HN and 166 without) and showed no significant difference between groups (RR = 1.06; 95%CI 0.41–2.75; *p* = 0.90; I^2^ = 0.0%; low certainty) (Fig. 10).

**Figure 11:**
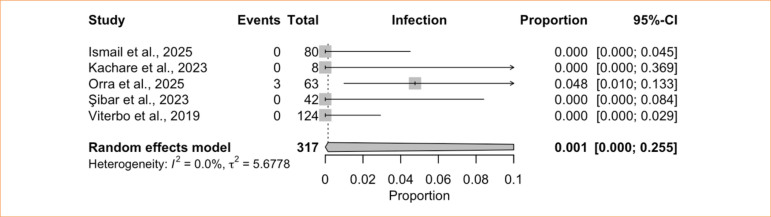
Proportional meta-analysis of infection: hemostatic net only.

Source: Elaborated by the authors.

**Figure 12:**
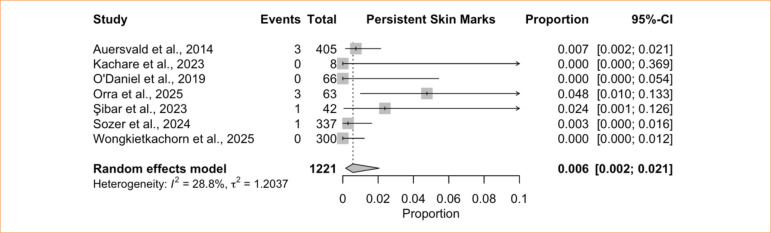
Proportional meta-analysis of skin marks: hemostatic net only.

Source: Elaborated by the authors.

The single-arm meta-analysis of infection included five studies^8,41,43,44,46^, with 317 patients treated with HN, and showed an incidence of 0.1% (95%CI 0.0–25.5%; I^2^ = 0%) (Fig. 11). For persistent skin marks, the single-arm meta-analysis of seven studies^6,28,41,42,44,45^ comprising 1,221 patients demonstrated an incidence of 0.6% (95%CI 0.2–2.1%; I^2^ = 28.8%) (Fig. 12).

The qualitative synthesis of patient-reported satisfaction included data from four studies. Two of these addressed only patient concerns regarding the disappearance of skin marks after HN application, whereas the other two reported overall satisfaction with the surgical outcome rather than satisfaction specifically related to the net, as presented in Table 2.

Finally, all but one^29^ study reported other adverse events. Occasional cases of ischemia, swelling, congestion, and transient neuromuscular disturbances were described among patients treated with HN. Table 2 provides a detailed overview of these additional events.

### Quality assessment

**Figure 13:**
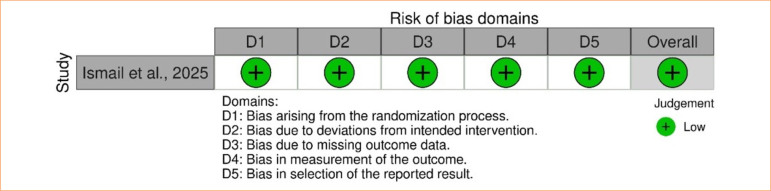
Traffic light plot: RoB 2.

Source: Elaborated by the authors.

Risk of bias was assessed using appropriate tools for each study design. For RCTs, the Cochrane Risk of Bias 2.0 tool^36^ was applied: the study by Ismail and Ghoraba^8^ demonstrated a rigorous methodological process, being evaluated as low risk of bias (Fig. 13).

**Figure 14:**
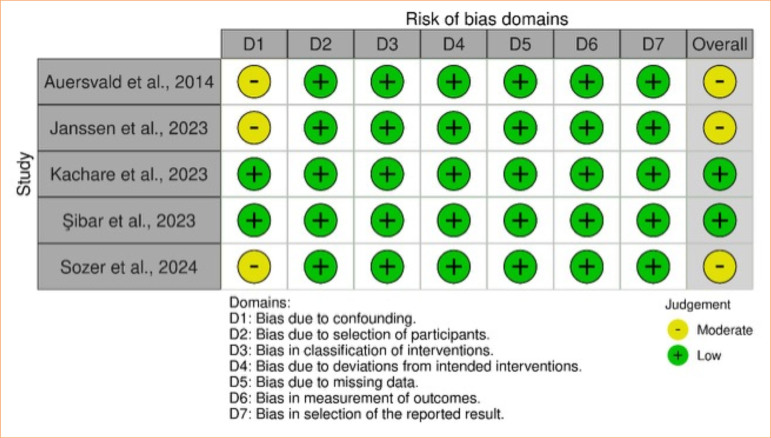
Traffic light plot: Robins-I.

Source: Elaborated by the authors.

Observational studies, such as retrospective cohorts and non-randomized prospective studies, were evaluated using the ROBINS-I tool^37^. Most showed moderate risk of bias^28,29,45^, mainly due to the absence of demographic data, while two studies, by Kachare et al.^41^ and Şibar et al^44^, were evaluated as low risk (Fig. 14).

**Figure 15:**
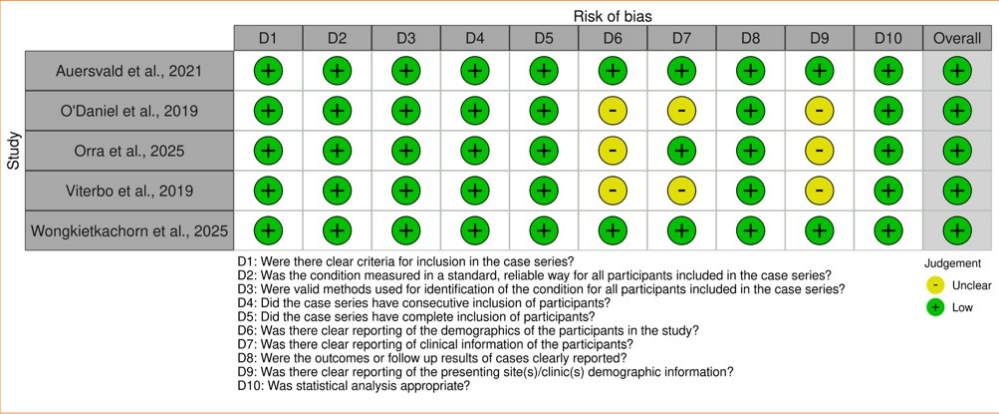
Traffic light plot: Joanna Briggs Institute Checklist for Case Series.

Source: Elaborated by the authors.

Case series studies were assessed using the JBI Critical Appraisal Checklist for Case Series^38^. All articles^6,40,42,43,46^ were considered as low risk of bias (Fig. 15).

### Certainty of evidence

The GRADE^39^ assessment is presented in Table 3. Because most of the included studies are non-randomized, the certainty of evidence could not be rated higher than low level of certainty.

**Table 3:** Certainty of evidence: GRADE.

Certainty assessment								N of patients			Effect		Certainty	Importance
N of studies	Study design	Risk of bias	Inconsistency	Indirectness	Imprecision	Other considerations		Hemostatic net	Drains		Relative (95%CI)	Absolute		
**Hematoma Controlled**														
5	Non-randomised studies	Not serious	Seriousa	Not serious	Not serious	None		3/897 (0.3٪)	31/645 (4.8٪)		**RR = 0.15** (0.01 to 3.20)	**41 fewer per 1.000** (from 48 fewer to 106 more)	⨁◯◯◯ Very low^a^	
**Skin necrosis controlled**														
4	Non-randomised studies	Not serious	Not serious	Not serious	Seriousb	None		3/593 (0.5%)	6/286 (2.1%)		**RR = 0.15** (0.04 to 0.58)	**18 fewer per 1.000** (from 20 fewer to 9 fewer)	⨁◯◯◯ Very low^b^	
**Seroma controlled**														
3	Non-randomised studies	Not serious	Not serious	Not serious	Not serious	None		8/188 (4.3٪)	7/166 (4.2٪)		**RR = 1.06** (0.41 to 2.75)	**Three more per 1.000** (from 25 fewer to 74 more)	⨁⨁◯◯ Low	
**Hematoma proportion**														
10	Non-randomised studies	Not serious	Not serious	Not serious	Not serious	None		14/2343 (0.6٪)	-		-	-	⨁⨁◯◯ Low	
**Skin necrosis proportion**														
7	Non-randomised studies	Not serious	Not serious	Not serious	Not serious	None		4/1062 (0.4٪)	-		-	-	⨁⨁◯◯ Low	
**Seroma proportion**														
7	Non-randomised studies	Not serious	Not serious	Not serious	Not serious	None		24/1,676 (1.4٪)	-		-	-	⨁⨁◯◯ Low	

95%CI: 95% confidence interval; RR: risk ratio; athere is an important heterogeneity between the studies; bonly one study contributed to the analysis. Source: Elaborated by the authors.

## Discussion

Despite a broad range of preventive strategies, including strict blood-pressure control, optimized pain management, local infiltration of vasoconstrictor, careful patient selection, and the use of quilting sutures, hematoma remains the most common postoperative complication of facial and cervical surgeries^17,18,20,21,32,33,47-52^. Quilting sutures, first described as early as 3400 BCE^26^ and introduced to plastic surgery in 1979^53^, gained wide acceptance after Baroudi and Ferreira^54^, demonstrated a significant reduction in seroma rates in abdominoplasty in 1998. By obliterating dead space, these stitches are hypothesized to lower the risk of both seroma and hematoma^6,8,26,28,29,32,33,40-46,55-58^. In 2003, Pollock and Pollock^55^ adapted this concept to rhytidectomy.

Seeking to avoid the potential skin irregularities associated with internal quilting, Auersvald and Auersvald^24,25^ introduced the HN in 2012, a continuous external suture resembling a fishing net, and reported a dramatic reduction in hematoma incidence from 14 to 0%. Since then, numerous studies have evaluated this technique with varying results.

Two systematic reviews have addressed quilting suture. However, these reviews show some limitations. Ballan et al.^59^ focused exclusively on quilting sutures in rhytidectomy and published before several pivotal studies were available. Caimi et al.^60^ incorporated more recent data but limited inclusion to facelifts and only four series using external HN, which restricts generalizability^61^. To address these gaps, we performed a comprehensive systematic review and meta-analysis of 11 studies on HN^6,8,28,29,40-46^ spanning both cervical and facial procedures, whether aesthetic or reconstructive.

Hematoma remains a critical concern after cervicofacial surgery, carrying risks that range from aesthetic compromise to life-threatening airway obstruction when located in the cervical region^17,23,47,62-64^. Preventing this complication is therefore paramount. In our analysis, the incidence of hematoma among patients treated with a HN was very low (0.6%), substantially below rates previously reported in patients without HN^23,47^ and consistent with prior evidence on internal quilting sutures^32,33^. This concordance suggests that the principal protective effect arises from mechanical obliteration of dead space rather than from whether the suture is external or internal.

In comparative analyses, the pooled estimate showed no significant difference in hematoma risk between patients with or without HN, except when the only randomized trial^8^ was excluded in LOO sensitivity analysis, which yielded a significant difference favoring net use. The dependence of statistical significance on the omission of this single RCT raises the possibility of publication bias in the non-randomized series. Such bias could not be formally assessed because funnel plots and Egger’s test are not recommended when fewer than 10 studies are available^34^. Overall heterogeneity for this endpoint was high and persisted despite LOO analysis. Given the negligible heterogeneity observed in the single-arm analysis of patients receiving the HN, we hypothesized that variability originated primarily in the control groups, in which hematoma incidence ranged from 0 to 14%. Meta-regression using control-group hematoma incidence as a moderator confirmed this hypothesis: the residual between-study variance (τ^2^) dropped to 0, indicating that this single factor fully accounted for the observed heterogeneity.

Skin necrosis is another recognized concern in cervicofacial surgery, as in any procedure involving skin undermining. Nevertheless, facial procedures generally carry a lower risk of ischemia or necrosis because of the region’s rich vascularity^65,66^. Theoretically, the external compression of a HN could compromise cutaneous blood supply or venous drainage, increasing the risk of ischemia or necrosis. However, recent evidence demonstrates that the net does not impair blood flow^67^, a finding consistent with our single-arm meta-analysis, which showed a very low incidence of necrosis (0.4%) with no heterogeneity. Our comparative meta-analysis even suggested a reduced risk of necrosis in the HN group, though this result must be interpreted cautiously because only one study contributed events, while the other three reported none in either arm.

Seroma after cervicofacial surgery is generally rare^47,68^, and our data confirm this. The single-arm analysis revealed a pooled incidence of 1.5% among patients treated with HN. Heterogeneity was initially high but dropped dramatically to 7.6%, and the incidence to 1.1%, after the exclusion of Ismail and Ghoraba^8^. This again raises concern about potential publication bias in the non-randomized studies and may also reflect differences in surgical techniques or in how seromas were diagnosed and reported.

Patient-reported outcomes (PROs) were poorly documented across the included literature. Only four of the 11 studies provided any PRO data: two simply noted nonspecific concerns regarding the disappearance of skin marks, and the other two reported overall satisfaction with the surgical result (gliding brow lift) rather than with the net itself. This paucity of PRO data is striking because PROs are widely recognized as essential in evaluating results of cervicofacial plastic surgery, whether aesthetic or reconstructive. Such measures capture dimensions not fully addressed by objective clinical metrics, such as satisfaction with appearance, psychosocial impact, quality of life, and overall well-being^69-73^. Given these gaps and the reported concerns about transient skin marks, we believe the development of a dedicated patient-reported satisfaction scale specific to the HN would substantially strengthen future evidence.

Infections are uncommon after face or neck lifts^74,75^, and our findings mirror this pattern, with a pooled incidence of only 0.1% in patients treated with HN. Early postoperative skin marks are common, as noted by Wongkietkachorn and Wongkietkachorn^6^, but approximately 90% of patients are free of marks by the end of the first postoperative month. Our single-arm analysis found a low incidence of persistent skin marks (0.6%), indicating that the HN provides hemostatic benefits without imparting a meaningful long-term aesthetic risk.

This review has some limitations. The scarcity of RCTs in plastic surgery was well recognized by Song and Chung^76^, and our meta-analysis included only one RCT^8^, which we rated as low risk of bias. For two outcomes, this trial acted as an outlier, raising concerns about potential publication bias among the non-randomized studies—which could not be formally evaluated in accordance with Cochrane^34^ guidance. Furthermore, substantial clinical and technical heterogeneity was observed across the included studies. Technical aspects such as suture material and caliber, spacing between stitches, precise anatomical placement, and dissection plane varied widely and were inconsistently reported, precluding systematic analysis. Although these factors—and the inherently operator-dependent nature of the technique—may influence outcomes, their impact could not be formally evaluated in the present analysis. Similarly, the distinction between minor and major adverse events could not be consistently accounted for, as most studies did not differentiate between events requiring surgical reintervention and those managed conservatively in an outpatient setting, including but not limited to hematoma.

Importantly, GRADE^39^ assessments indicated low to very low certainty for all analyzed outcomes, reflecting the predominance of non-randomized evidence, and the heterogeneity introduced into the controlled meta-analyses by historical control groups in some studies, as demonstrated by our meta-regression. Operative time was infrequently reported, and the potential impact of the hemostatic net on surgical duration remains unclear, warranting further investigation in future studies. Finally, while generally safe, the technique is not without inherent risks: all but one of the included studies documented adverse events beyond those formally analyzed, such as transient ischemia, congestion, swelling, and neuromuscular disturbances, likely reflecting the intrinsic challenges of an external quilting method applied without direct visualization of underlying structures and inevitably exerting some degree of skin compression, even when tension is low.

## Conclusion

The HN appears safe, with consistently low rates of hematoma, skin necrosis, and other complications. However, proving its superiority over standard surgery remains challenging due to the overall low certainty of evidence and the heterogeneity between the analyzed surgical procedures. Moreover, the lack of PROs highlights the need to develop dedicated PRO instruments to assess satisfaction and quality-of-life impacts in patients undergoing cervicofacial procedures with HN.

## Data Availability

All data sets were generated or analyzed in the current study.
